# The contribution of cell surface FcRn in monoclonal antibody serum uptake from the intestine in suckling rat pups

**DOI:** 10.3389/fphar.2014.00225

**Published:** 2014-10-07

**Authors:** Philip R. Cooper, Connie M. Kliwinski, Robert A. Perkinson, Edwin Ragwan, John R. Mabus, Gordon D. Powers, Haimanti Dorai, Jill Giles-Komar, Pamela J. Hornby

**Affiliations:** ^1^Biologics Research, Janssen R&D – Johnson & Johnson, Biotechnology Center of ExcellenceSpring House, PA, USA; ^2^Biologics Pharmacology and Toxicology, Janssen R&D – Johnson & Johnson, Biotechnology Center of ExcellenceSpring House, PA, USA

**Keywords:** FcRn, IgG, uptake, bioavailability, cell surface receptor

## Abstract

The neonatal Fc receptor (FcRn) in intestinal epithelium is the primary mechanism for transfer of maternal immunoglobulin G (IgG) from suckled milk to serum; but the factors contributing to the rapid uptake of IgG are poorly understood. These studies help to determine the contribution of cell surface FcRn in IgG uptake in 2-week-old rat pups by varying local pH and binding conditions. Variants of a human wild-type (WT) IgG monoclonal antibody (mAb WT) were assessed for binding affinity (K_D_) to rat (r)FcRn at pH 6.0 and subsequent off-rate at pH 7.4 (1/s) by surface plasmon resonance. Selected mAbs were administered intra-intestinally in isoflurane-anesthetized 2-week rat pups. Full length mAb in serum was quantified by immunoassay, (r)FcRn mRNA expression by reverse transcription polymerase chain reaction, and mAb epithelial localization was visualized by immunohistochemistry. After duodenal administration, serum levels of mAb variants correlated with their rFcRn off-rate at pH 7.4, but not their affinity at pH 6.0. The greatest serum levels of IgG were measured when mAb was administered in the duodenum where rFcRn mRNA expression is greatest, and was increased further by duodenal administration in pH 6.0 buffer. More intense human IgG immunostaining was detected in epithelium than the same variant administered at higher pH. These data suggest an increased contribution for cell surface receptor. We conclude that, in the neonate duodenum, receptor off-rates are as important as affinities for FcRn mediated uptake, and cell surface binding of IgG to rFcRn plays contributes to IgG uptake alongside pinocytosis; both of which responsible for increased IgG uptake.

## INTRODUCTION

Neonatal Fc receptors bind to the Fc portion of IgGs and function in infant mammalian physiology to facilitate transport of IgG from the placenta to the fetus, and from suckled maternal milk into the neonatal circulation (Brambell, 1966, 1969; Rodewald and Kraehenbuhl, 1984). Beyond mammalian infancy, FcRn is still expressed in the intestine at much lower levels (Hornby et al., 2013), but most commonly found constitutively expressed in the endothelium, and is responsible for the increased half-life of IgGs in the circulation (Roopenian and Akilesh, 2007).

Endothelial cells take up surrounding fluids and solutes (including all Igs), by pinocytosis to form intra-cellular vesicles to collect nutrients for the cell or to breakdown unwanted particles in the circulation. Within these endosomes, conditions become slightly acidic (pH 6.0) and favorable for the interaction between FcRn molecules and the Fc portion of IgGs. Unbound IgGs, all IgAs and IgMs, and other unwanted materials are broken down by lysosomes. FcRn bound IgGs are recycled back to the cell surface, disassociate from the FcRn in neutral (pH 7.4) conditions and released back into the circulation (Brambell, 1966, 1969; Raghavan et al., 1995).

In contrast to the endothelial cells, the FcRn–IgG complex is transcytosed through the intestinal epithelium cell and is released on the basolateral side, disassociated and the IgG and is released into the lymphatic system, where it enters the circulation via the thoracic lymph nodes (Swartz and Skobe, 2001; Kliwinski et al., 2013). The mechanism of internalization of IgG in epithelial cells remains quite controversial as to whether pinocytosis and cell surface FcRn receptors both contribute (Benlounes et al., 1995; Mohanty et al., 2013). Evidence suggests that FcRn receptors are located on the cell surface of enterocytes (Israel et al., 1997; Hornby et al., 2013); suggesting that IgG internalization could occur by cell surface binding and uptake in epithelial cells.

The intestinal system of neonates is adapted to these challenges by having a reduced level of secreted protease enzymes, an under-developed intestinal epithelial layer, and increased expression of FcRn on the luminal side of intestinal epithelial cells (Udall et al., 1981; Norin et al., 1986; Telleman and Junghans, 2000). Therefore, neonates, such as suckling rat pups, may serve as a suitable mechanistic *in vivo* model for IgG transcytosis (Rodewald and Kraehenbuhl, 1984; Benlounes et al., 1995; Martin et al., 1997).

We previously showed that Tg276 transgenic mice that express human FcRn in the intestinal mucosa do not functionally transport human IgG after intestinal administration (Kliwinski et al., 2013). When human IgG was administered to the small intestine of 2-week-old suckling rat pups, approximately 80% of the uptake was FcRn-dependent while the remaining ∼20% was FcRn-independent and non-receptor mediated (Kliwinski et al., 2013).

The present study aims to further characterize the pharmacological interactions between IgG and FcRn that contribute to the rapid uptake of IgG in the neonatal rat, including FcRn affinity and off-rates, pH-dependence, the effect of differential intestinal administration sites, and aims to increase evidence that cell surface receptors have a role.

## MATERIALS AND METHODS

### DETERMINING THE AFFINITY AND OFF-RATES OF HUMAN IgG VARIANTS TO RAT FcRn

The mAbs used in this study were a recombinant chimeric human IgG1 monoclonal antibody specific for human respiratory syncytial virus (RSV), also known as B21M. The WT and FcRn binding affinity variants (H435A, N434A, and N434Y) with mutations at the 434 and 435 amino acid positions (EU numbering; Firan et al., 2001; Yeung et al., 2009; Deng et al., 2012) were tested for binding affinity to rat FcRn. In addition to determining the affinity of human mAbs to rat FcRn at pH 6.0 and 7.4 we developed a method to determine the off-rate of pre-bound (at pH 6.0) mAb after the buffer conditions were changed to pH 7.4.

#### GLC biosensor chip conditioning and Rat FcRn immobilization

A ProteOn XPR36 GLC biosensor chip (Bio-Rad, Hercules, CA, USA) was preconditioned with 0.5% sodium dodecyl sulfate (SDS), 50 mM NaOH and 100 mM HCl in both the vertical (ligand) and horizontal (analyte) channels. Following equilibration with PBS-TE running buffer (20 mM Na-phosphate, 150 mM NaCl, 0.005% Tween-20, 3 mM EDTA, pH 6.0), FcRn was immobilized using an amine coupling kit at a temperature of 25°C and a flow rate of 30 μl/min. All channels were activated with a mixture of EDC [1-Ethyl-3-(3-dimethylaminopropyl) carbodiimide] (0.2 M) and sulfo-NHS (0.05 M) at 30 μl/min for 4 min, followed immediately by immobilization of rat FcRn (3 μg/mL in 10 mM sodium acetate pH 4.5) over channels 1 and 2 at 30 μL/min for 5 min. The reference channel was treated identically without injection of FcRn. All channels were then blocked for 5 min with an injection of 1 M ethanomine-HCl (pH 8.5). This method resulted in rat FcRn coupled at response levels of 193 and 161 RU (1 RU = 1 pg protein/mm^2^) in channels 1 and 2, respectively.

#### Analysis of affinity between rat FcRn and IgG variants at pH 6.0

Following immobilization, the four IgG1 (WT, N434A, N434Y, and H435A) mAb variants were diluted with running buffer formulated at pH 6.0. Each mAb was tested at five concentrations in duplicate using a fivefold dilution series. The five concentrations of each analyte were injected simultaneously at a flow rate of 60 μL/min for a 1 min association phase which was followed by a 3 min dissociation phase. The surface was regenerated prior to each subsequent mAb and tested with two injections of sodium phosphate (pH 8.0) followed by one injection of running buffer (pH 6.0). Affinity analysis and kinetic constants were calculated from the sensorgrams using the bivalent analyte model of the ProteOn XPR36 software.

#### Analysis of off-rate between rat FcRn and IgG variants at pH 7.4 using the ProteOn co-injection mode

Following immobilization, the four IgG1 mAb variants were diluted with running buffer formulated at pH 6.0. Each mAb was tested at five concentrations in duplicate using a fivefold dilution series. The five concentrations of each analyte were injected simultaneously at a flow rate of 60 μL/min for a 1 min association phase immediately followed by a co-injection step in which running buffer formulated at pH 7.4 (with no IgG present) was injected for a 3 min dissociation phase. The surface was regenerated prior to each subsequent mAb tested with two injections of sodium phosphate (pH 8.0) followed by one injection of running buffer (pH 6.0). Off-rate analysis was evaluated using ProteOn XPR36 software.

***Intra-intestinal dosing.*** Rat pups, 12–17 days-old (male or female), were kept with their dams in plastic filter-topped cages until 30 min prior to the start of each experiment. The animals were placed in a chamber for induction of anesthesia with isoflurane (initially 2–4%) and then removed and placed supine on a surgical pad maintained at 37°C with a nose cone for maintenance of anesthesia (2% isoflurane). Animals were prepared for the surgical procedure, which consisted of a laparotomy followed by exteriorization of the stomach and start of the small intestine using minimal disturbance. A bolus injection of test article (1 mg/kg) was made into either the proximal small intestine (duodenum) or the distal small intestine (ileum), depending on the study, via a 27 g needle. A retro-orbital blood draw of approximately 100 μL 5 min after test article administration, and a terminal intracardiac withdraw was performed at 90 min. All blood samples were allowed to stand for at least 30 min, but no longer than 1 h, centrifuged at 3500 rpm for 10 min and the serum separated and stored at -80°C until analyzed. The entire intestine was also removed and cleaned of mesenteric attachments and laid out to its full length without stretching. The small intestine divided into thirds, and the proximal colons (∼3 cm from the cecum) were collected from each rat pup. The tissue plus luminal contents were added to individual 2-mL tubes containing 250 μL of rat serum with protease inhibitor cocktail at a final concentration of one tablet/10 mL and kept on ice for at least 30 min. All samples were centrifuged at 3500 rpm for 10 min and the supernatant separated and stored at -80°C until valuated. Care was taken to perform the surgical procedure following aseptic techniques and a surgical plane of anesthesia was maintained throughout the entire procedure. All animal studies were performed in accordance with the Federal Animal Welfare Act and protocols were approved by the Institutional Animal Care and Use Committee (IACUC) at Janssen R&D.

***Intact IgG analysis.*** Full length IgG antibodies were quantified using the Meso Scale Discovery (MSD) electrochemiluminescent assay as previously described (Hornby et al., 2013; Kliwinski et al., 2013). The mAbs used in this study were a recombinant chimeric human IgG1 monoclonal antibody specific for human RSV, also known as B21M. An anti-idiotypic antibody specific for the anti-RSV IgGs was labeled with sulfo-NHS-LC-biotin and used as a capture antibody. A pan anti-human IgG1/IgG2 antibody labeled with MSD Sulfo-TAG NHS Ester was used as the detection antibody. A standard curve for the anti-RSV IgGs in neat rat serum was observed over the range of 50,000 pg/mL–50 pg/mL. The lower limit of quantification (LLOQ) was 0.5 ng/mL using 25 μL of neat rat serum. Serum and intestinal content levels of intact IgG antibodies are reported as mean ± SEM (ng/mL).

***FcRn RT-PCR.*** Mucosal scrapings from rat pup duodenum, ileum, and proximal colon were snap frozen immediately following sample collection. Scrapings were placed into FastPrep lysing tubes with 500 μL lysis buffer and rapidly shaken for 60 s. The homogenous sample (350 μL) was used to make RNA using the QIAGEN RNAeasy Kit (Valencia, CA, USA). RNA concentration was confirmed using the NanoDrop spectrophotometer (Invitrogen, Carlsbad, CA, USA). RNA integrity was established using the RNA Nano 6000 Kit and read on an Agilent 2100 Bioanalyzer (Santa Clara, CA, USA). RNA was stored at -80°C. The cDNA was made using a reverse transcription kit from Applied Biosystems (Carlsbad, CA, USA) from approximately 0.3–1.0 μg of RNA and stored at -20°C. Reverse transcription polymerase chain reaction (RT-PCR) samples were prepared in triplicate with the following: 2× Taqman Universal PCR Master Mix (10 μL), distilled water (8 μL), 20× primer and probe mix (1 μL) and cDNA (1 μL; 20–50 ng). The cycle threshold (Ct) was normalized to rat housekeeping gene, hypoxanthine phosphoribosyltransferase (HPRT). The intestinal segment with the lowest Ct value relative to the housekeeping gene was arbitrarily used as the comparator for the remaining three gut sections (proximal colon for rat). A fold change with respect to the proximal colon was performed using a delta–delta Ct method which involved normalization to a housekeeping gene and fold change with respect to a particular gut section.

The primer and probe sets used were: Rat B2M, Rn00560865, Rat FcRn, Rn00583712, Rat HPRT, Rn01527840, and Rat GAPDH, Rn99999916 (Applied Biosystems).

***Immunohistochemistry.*** Following intra-duodenal administration of IgG variants, a 1-cm section of the duodenum was removed and laid flat between two pieces of biopsy sponge and fixed in 10% neutrally buffered formalin (NBF). Tissues were exposed to an increasing alcohol gradient and 5-micron sections were cut from each tissue block sample and de-waxed. A steam heat-induced epitope recovery pretreatment system (SHIER2) was used followed by a goat block and the primary antibody, human IgG1 (1 μg/mL; 1 hr; Epitomics). A biotinylated goat anti-rabbit IgG was then incubated with the sections, followed by a hydrogen peroxide block and the ABC (avidin/biotin complex) reagent. The 3,3′-diaminobenzidine (DAB) chromogen was added followed by a hematoxylin counterstain and dehydration. Specimens were cover-slipped with glass and Permount^TM^. The above procedures were completely automated using the TechMate 500 (BioTek Solutions). Positive staining was indicated by the presence of a brown chromogen (DAB-HRP) reaction product. Hematoxylin counterstaining provided a blue nuclear stain to assess cell and tissue morphology. Representative images were obtained with an Olympus Microfire digital camera (M/N S97809) attached to an Olympus BX60 microscope. Images were graded on a scale of 0–3 by a blinded investigator.

## RESULTS

### AFFINITY AND OFF-RATES OF IgG1 VARIANTS TO RAT FcRn AT pH 6.0 AND 7.4

**FIGURE 1:**
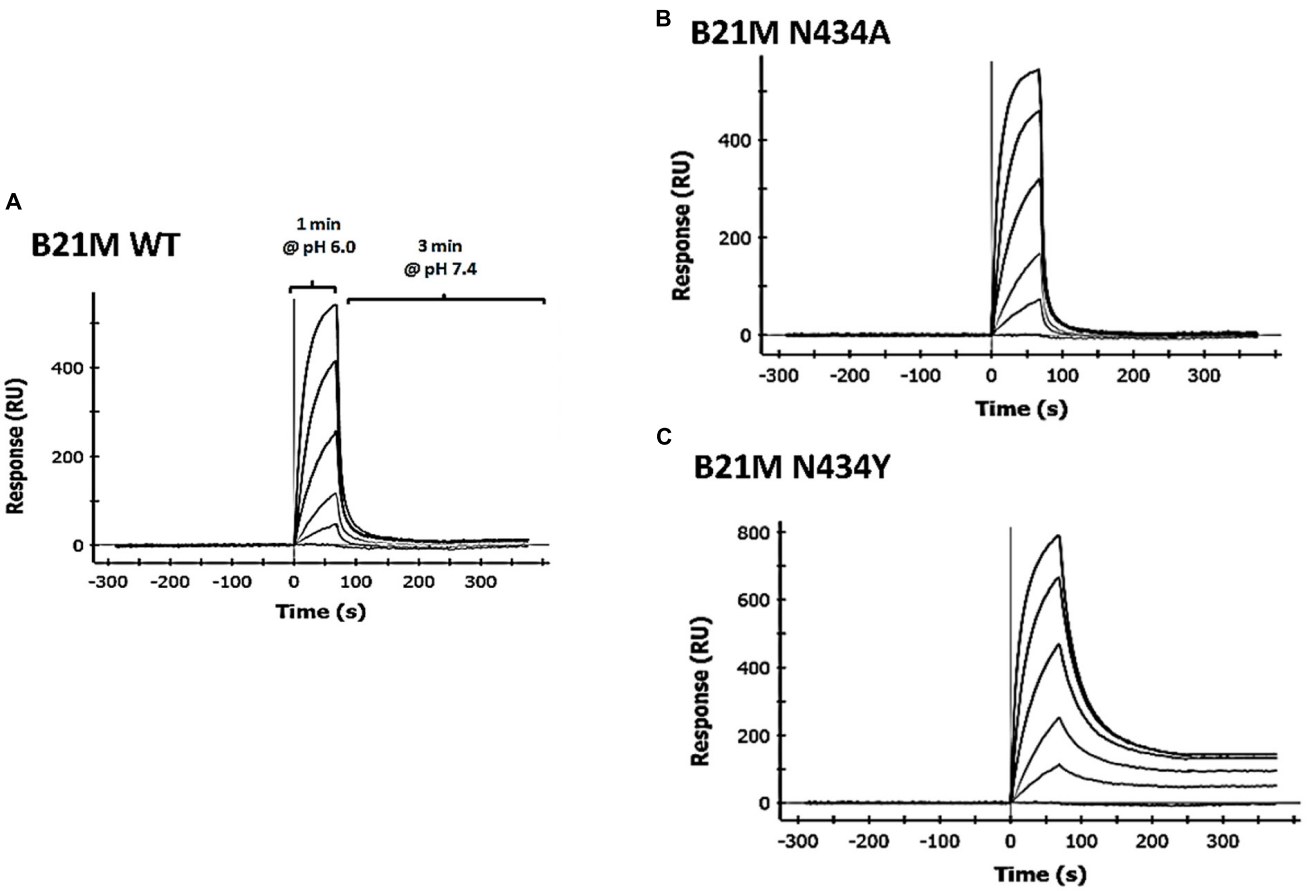
**Surface plasmon resonance (SPR) analysis using the ProteOn XPR36 in co-injection mode IgG1 variants, **(A)** wild-type (WT); **(B)** N434A or **(C)** N434Y were diluted 1:5 in a concentration series from 1000 to 1.6 nM, injected over rat FcRn for 1 min in PBS-TE (pH 6.0) and followed immediately by a co-injection of PBS-TE (pH 7.4) without mAb for 3 min**.

We developed methodology to mimic the effect from pH change of pH 6.0 to pH 7.4 on IgG-FcRn binding, which enabled determination of the off-rates of pre-bound mAb. Four human IgG1 variants were passed over the rat-FcRn pre-coated ProteOn chip in separate lanes for one min at pH 6.0 at five different concentrations between 1.6 and 1000 nM in duplicate. Sufficient time was allowed for Fc-FcRn association to determine affinities after which the media was changed to PBS-TE (pH 7.4) without the IgG variants to determine FcRn off-rates. Affinities to FcRn in increasing order were WT: 169 ± 8.8 nM, N434A: 77 ± 0.2 nM, and N434Y: 27 ± 3.6 nM (**Table 1**). The H435A variant did not bind to FcRn at any concentrations up to 1000 nM (data not shown) at either pH 6.0 or 7.4. The WT and N434A variants completely disassociated from FcRn at a faster rate ( respectively) than the N434Y variant at pH 7.4, which still had affinity to FcRn at this pH (**Figure 1C**).

**Table 1:** Affinities and off-rates of IgG1 variants to FcRn.

	Affinity to rat FcRn (nM)		pH shift (6–7.4)
IgG1 variant	pH 6.0	pH 7.4	rFcRn off-rate 1/s (s)
WT	169 ± 8.8	–	0.07 ± 0.0004
N434A	77 ± 0.2	–	0.09 ± 0.001 (11 s)
N434Y	27 ± 3.6	1240	0.02 ± 0.0008 (50 s)
H435A	–	–	–

### SERUM LEVELS OF IgG CORRELATE WITH RAT FcRn pH 6.0 TO 7.4 OFF-RATES

**FIGURE 2:**
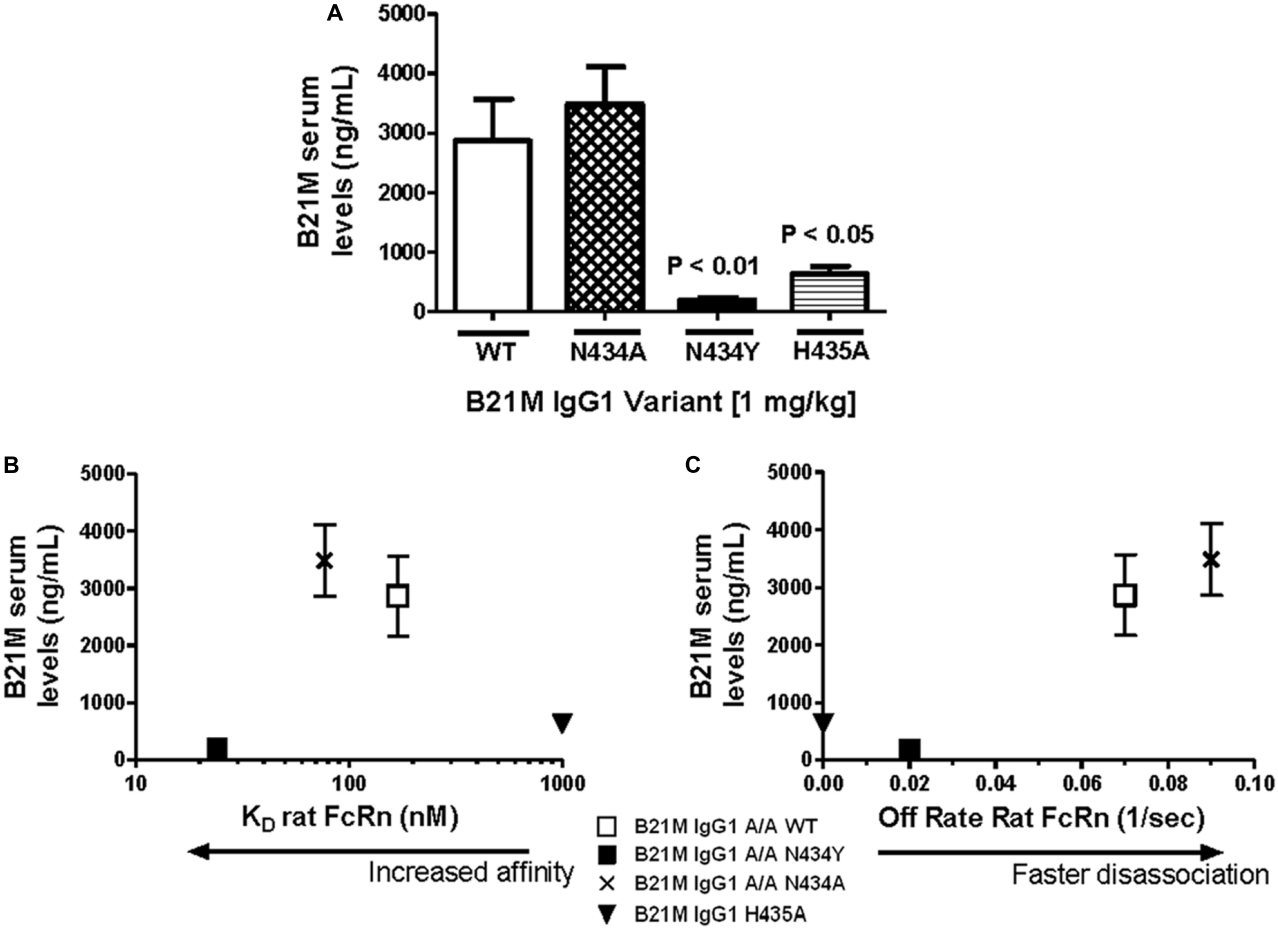
**Serum levels of IgG correlate with rat FcRn pH 6.0 to 7.4 off-rates: (A) Serum levels (ng/mL) of IgG1 variants (1 mg/kg) 90 min after intra-duodenal administration in sucking rat pups (n = 5) were highest for N434A andWT and low as expected for H435A, with N434Y similar to H435A.** One-way Analysis of variance (ANOVA) statistics were carried out followed by a Bonferroni’s multiple comparison test versus WT. Serum levels were plotted against **(B)** Kd affinity constants (nM) to rat FcRn at pH 6.0 (r 2 = 0.43; P = 0.54); and **(C)** dissociation from FcRn (1/s) at pH 7.4. after pre-binding at pH 6.0 (r 2 = 0.99; P = 0.07). Key: □WT; ▪ N434Y; × N434A; and ▼ H435A. The H435A has low > 1000 nM binding affinity to FcRn, and its points are only added to **(B)** and **(C)** to show the trend. All statistics carried out excluded the H435A points to avoid bias.

Serum samples at 5 and 90 min following the administration of either IgG1 WT, N434A, N434Y, or H435A (1 mg/kg) into the duodenum of sucking rat pups were analyzed for full length IgGs. Quantities of all IgG1 variants were below the LOQ after 5 min and so served as a baseline reading. The IgG1 N434A variant serum levels at 90 min of 3483 ± 624 ng/mL (**Figure 2A**) was trending slightly higher than levels of the WT variant at 2864 ± 700 ng/mL, followed by the H435A variant at 630 ± 131 ng/mL. Surprisingly, the serum level of N434Y after 90 min (195 ± 39 ng/mL) was low despite previously being shown to have the greatest affinity to rat FcRn (27 nM). Increasing the length of time after duodenal administration to 180 min increased the serum levels of N434A, WT, H435A, and N434Y to 6040 ± 797, 5840 ± 792, 1154 ± 351, and 363 ± 53 ng/mL, respectively, but did not change the relative amounts absorbed by each variant. There didn’t appear to be a relationship between serum levels detected and rat FcRn binding affinity, since the highest FcRn affinity variant, N435Y, had the lowest serum levels (**Figure 2B**). However, there was a trend between serum levels and rat FcRn off-rates at pH 7.4 in that the faster the disassociation from rat FcRn at pH 7.4, the greater the levels detected in the serum (**Figure 2C**).

### SERUM LEVELS OF IgG CORRELATE WITH RAT FcRn mRNA EXPRESSION

**FIGURE 3:**
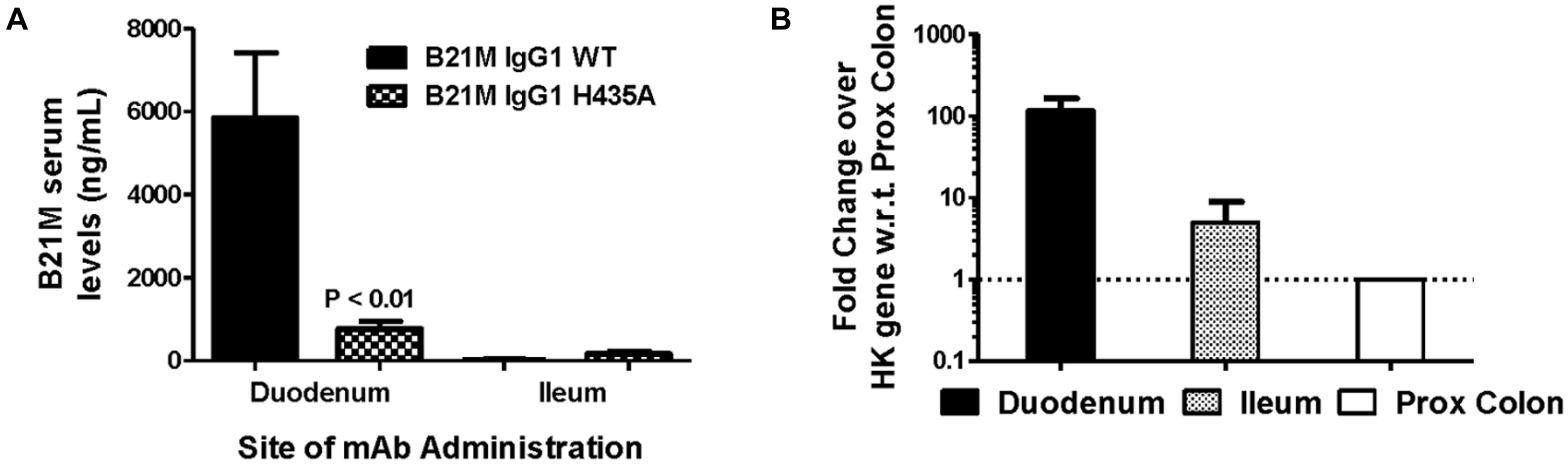
**Greatest serum levels of IgG measured when administered at site with greatest rat FcRn mRNA expression: **(A)** Serum levels of the WT mAb were measured after 90 min following administration at the proximal small intestine (S.I; duodenal region) and at the distal S.I (ileum region) in suckling rat pups (*n* = 6).** A one-way ANOVA followed by Bonferroni’s multiple comparison test was carried out versus respective WT. **(B)** mRNA expression of rat FcRn, assessed using reverse transcription polymerase chain reaction (RT-PCR), from mucosal scrapings of proximal and distal small intestine, and proximal and distal colon. Data shown is fold change over proximal colon which had the lowest level of FcRn mRNA).

The WT IgG1 with a Kd value of 169 nM and the low FcRn affinity variant, H435A, (Kd > 1 μM) were administered (1 mg/kg) at the site of the proximal (duodenum) or the distal (ileum) small intestine of suckling rat pups. Retro-orbital and cardiac bleeds were taken at 5 and 90 min following administration. Serum levels of the WT mAb were greater when administered at the proximal small intestine compared to the distal small intestine (5833 ± 1561 ng/mL versus 26.9 ± 12.5 ng/mL). The serum levels of WT after proximal small intestine administration were greater than the H435A variant administered to the same site (772 ± 178 ng/mL; *P* < 0.01). When the variants were administered at the distal small intestine, there was no difference between the WT and H435A variant (**Figure 3A**).

Reverse transcription polymerase chain reaction was used to quantify the relative FcRn mRNA expression in the different regions of the rat intestinal mucosa. Greater amounts of rat FcRn were detected in the proximal and mid small intestine (**Figure 3B**), compared to the distal small intestine and proximal colon.

### THE EFFECT OF BUFFER pH ON SERUM UPTAKE OF HIGH AND LOW RAT FcRn-AFFINITY IgG VARIANTS

**FIGURE 4:**
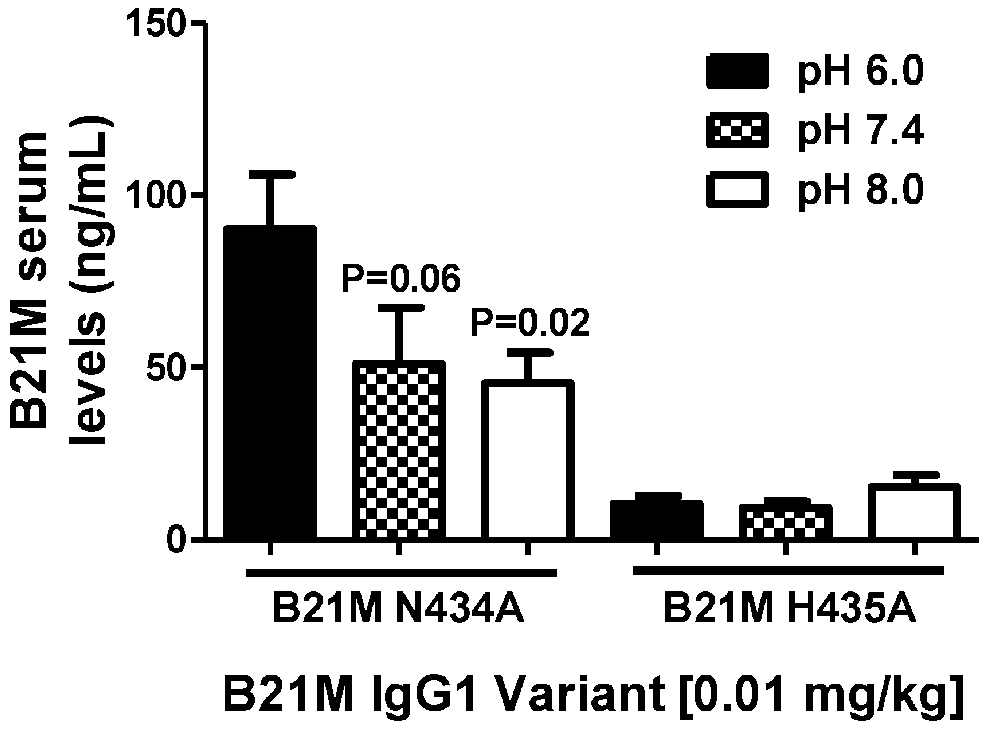
**The effect of pH (pH 6.0, 7.4, and 8.0.) of high and low FcRn-affinity IgG variants on serum uptake: Serum levels (ng/mL) at 90 min after administration of N434A tended to be higher following intra-duodenal administration (0.01 mg/kg) in suckling rat pups (*n* = 6).** There was no difference in the serum levels of H435A administered in different pH conditions. One-way ANOVA Statistical tests carried out compared to N434A pH 6.0.

Unlike endothelial cells, it is thought that epithelial cells express FcRn on the cell surface. Suckling rat pups were administered the rat FcRn binding variant, N434A, or the non-binding variant, H435A, to the duodenum (0.01 mg/kg). The variants were administered in pH conditions of 6.0, 7.4, and 8.0. Serum was collected after 90 min and full length mAb levels were quantified. The variant with the greater rat FcRn binding affinity variant was affected by buffer pH in that greater amounts of the variant were measured in the serum when administered at pH 6.0 (90.2 ± 15.1 ng/mL), compared to levels detected in the serum when administered at pH 7.4 or 8.0 (*P* = 0.06 and *P* = 0.02, respectively; **Figure 4**).

**FIGURE 5:**
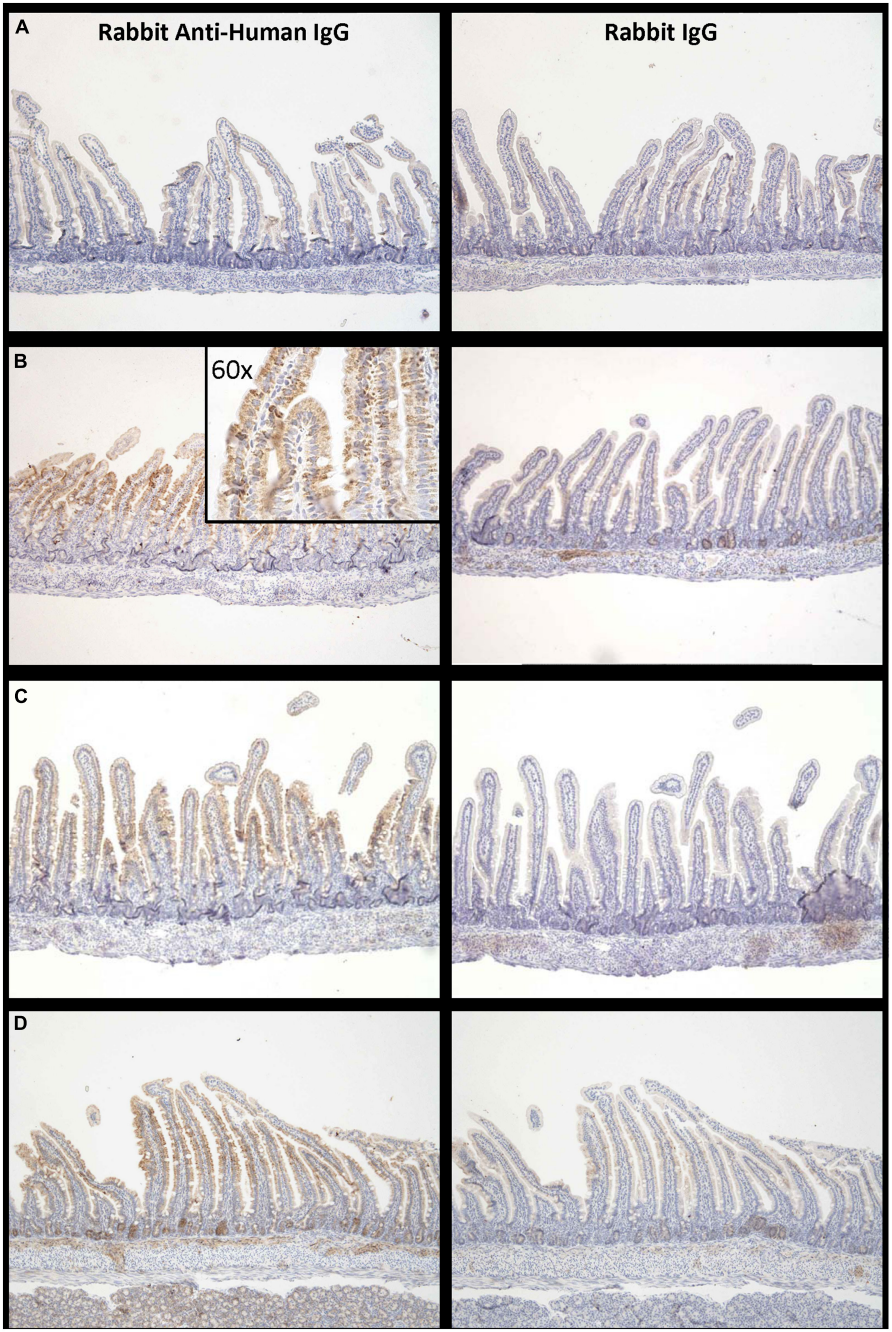
**Increased levels of IgG bound to enterocytes at pH 6.0 compared to pH 8.0: Representative examples of IgG –immuno-reactivity in enterocytes of suckling rat pup proximal small intestine 90 min after administration of (A) PBS, (B) IgG1 N434A at pH 6.0, (C) IgG1 N434A at pH 8.0, and (D) IgG1 H435A at pH 8.0.** Images on the left-hand side are using rabbit anti-human IgG, and the images on the right are using the anti-rabbit IgG to show specificity. Images are at 10× magnification with the exception of the inserted image in **(B)** which is 60× magnification.

Proximal small intestine tissue was collected at 90 min. Anti-human IgG1 immunostaining was noted at the brush border epithelium in the proximal small bowel of IgG1-infused rats. Representative images of immunostaining are shown for each of the three groups (**Figure 5**) illustrating that the most marked human IgG immunostaining in enterocytes was visualized when the high FcRn binding affinity variant was administered at pH 6 (**Figure 5B** compared to **Figure 5C**). Immunostaining after administration of the low FcRn binding variant at pH 8.0 was also qualitatively less than that visualized with the FcRn binding variant administered in pH 6.0 buffer. A representative image from the control group that was infused with PBS, did not show any human IgG1 immuno-reactivity and was similar to rabbit IgG control antibody staining (**Figure 5A**). Semi-quantitative immunostaining scoring (0 = no staining, and 3 = intense staining) performed by a histologist blinded to the treatment supports the pattern of staining shown in the representative images from each group. Tissue from animals infused with high affinity FcRn binding variant at pH 6.0 had an average immuno-reactivity score of 1.5 (**Figure 5B**) while those infused at pH 8.0 had an immuno-reactivity score of 1.167 (**Figure 5C**) and tissue from animals infused with low FcRn binding affinity variant at pH 8.0 had an average immune-reactivity score of 0.5 (**Figure 5D**).

## DISCUSSION

The present study in neonatal rat intestine demonstrated a role for both intracellular and cell surface FcRn in the uptake and transcytosis of IgGs. Consistent with our previous study (Kliwinski et al., 2013), IgG affinity to FcRn is important for intestinal to serum uptake; additionally, we now demonstrated that mAb variant off-rates (K_o_), the location of IgG administration, and pH conditions were also important for FcRn-dependent uptake. Serum uptake was increased, when: (1) using variants with the fastest FcRn off-rates at pH 7.4; (2) administered into the duodenum where the greatest amount of FcRn is located; and, (3) local conditions are at pH 6.0 to increase the likelihood of cell surface FcRn binding and internalization.

We previously demonstrated that transgenic mice incorporated with human FcRn did not functionally transport human IgG from the intestine into the circulation (Kliwinski et al., 2013). We therefore used the suckling rat pup model to demonstrate the mechanistic pharmacological functions of FcRn and IgG. Four human IgG variants whose association with human FcRn has been established (Kuo and Aveson, 2011) were engineered for use in the current study: WT, H435A, N434A, and N435Y. Previously published studies have shown that a rat FcRn exhibited specificity for human IgG, but failed to bind to mouse IgG (Kuo et al., 2009); thus, the affinities of the human (h)IgGs to rat FcRn were determined. The rank order of affinity was N434Y >> N434A > WT >> H435A. Following intra-duodenal administration, the rank order of serum levels was N434A > WT >> H435A > N434Y. It was surprising that the variant with the greatest affinity toward rat FcRn was the least measured in the serum, when it was expected that greater FcRn affinity would yield greater serum uptake. Quantities of all the variants in the small intestine after 90 min were comparable suggesting that there was no unforeseen increase in degradation in the duodenum. The N434A variant has been shown to have a greater affinity to FcRn, than the WT, across many species (Deng et al., 2010), resulting in an increased half-life in the circulation. However, some studies (Gurbaxani et al., 2006; Datta-Mannan et al., 2007a,b) have not shown a correlation between FcRn affinity and half-lives, suggesting that other pharmacological properties, such as off-rates, are important.

The off-rate of pre-bound human IgG to rat FcRn at pH 6.0 was calculated when the buffer was increased to pH 7.4. The rank order of off-rates was N434A > WT >> N434Y, which correlated with intact IgG levels in the serum. The off-rate at pH 7.4 of the H435A variant could not be measured as it had no calculated binding at pH 6.0. The lower than expected levels of the N434Y variant in the serum remains bound within the endosome due to its slow disassociation from, and affinity toward FcRn at pH 7.4.

In a previously published *ex vivo* study using rat pup intestinal segments mounted in Ussing-type flux chambers, IgG transcytosis from the mucosal side was greater in segments from the proximal than distal small intestine (Benlounes et al., 1995); however, the mechanisms responsible for this difference were not elucidated. In the present study we determined that FcRn mRNA expression is 100-fold higher in the proximal small intestine than the colon, agreeing with previously published studies showing decreasing levels of FcRn mRNA down the small intestine (Martin et al., 1997). The relative measure of FcRn mRNA was based on mucosal samples rather than full thickness segments; therefore, the fold changes in different regions are reflective of epithelial FcRn mRNA expression not FcRn expression in other cell types such as endothelial and lymphocytes. Consistent with the expression data, significantly greater levels of IgG WT were seen in the serum following administration into the proximal small intestine compared to the H435A variant. These results, and those from a previous *in-vivo* studies, (Martin et al., 1997; Kliwinski et al., 2013) confirm that an FcRn-dependent mechanism accounts for a majority of IgG uptake in the proximal small intestine. Also, after administration of IgGs into the distal small intestine, where FcRn mRNA expression was much lower, the serum levels of WT were <30 ng/mL and were similar to levels of the low FcRn binding affinity variant, H435A suggesting that non-FcRn dependent uptake was primarily contributing to the serum levels after ileum dosing. Regional uptake in isolated segments of rat pup small intestine in Ussing chambers was attempted in the present study; however, full-thickness small intestine did not result in any IgG on the basolateral side 90 min after apical application and it was too technically challenging to muscle-strip these fragile tissues without damaging the integrity of the barrier (detected by FITC-dextran passing across the chamber and the poor histological appearance of paraffin imbedded H&E stained sections).

There is a continuing debate as to whether entereocytes express FcRn on their cell surface, and if the cell surface expression contributes to IgG uptake. An *ex vivo* study determined that local pH conditions of 6.0 did not increase the uptake of IgG in neonatal proximal small intestine, therefore suggesting that cell surface receptors were not present and did not amplify cellular uptake (Benlounes et al., 1995). An *ex vivo* study concluded that there was no FcRn present on the jejunum enterocyte surface of weaned rats, as well as limited FcRn present on neonatal jejunum with most being intra-cellular (Berryman and Rodewald, 1995). However, *in vitro* studies have shown, by flow cytometry, that FcRn is located on a majority of human intestinal caco-2 cells; and cell surface IgG binding occurs with a high-affinity FcRn-binding IgG (Hornby et al., 2013). Using transgenic FcRn^+/+^ and FcRn^-/-^ mice strains, one study demonstrated increased amounts of IgG in the cytoplasm of the knock-out strain verses the over-expressing concluding Brambell’s hypothesis that receptor-ligand binding occurs in the cell with no role of FcRn cell surface receptor binding (Mohanty et al., 2013). This result was unexpected, but could be explained by our previous transgenic studies indicating that this *in vivo* model may not be ideal to assess the function and role of entereocyte FcRn (Kliwinski et al., 2013). The study did indicate that FcRn is required for exocytosis from the cell (Ober et al., 2004; Mohanty et al., 2013), and with the assumption that endosome exocytosis occurs both as a recycling and transcytosis pathway in the enterocyte (Dickinson et al., 1999), it is totally plausible that vacant FcRn would be present on the apical cell surface for a limited time. Indeed, the authors who noted limited FcRn present in the neonatal jejunum (Berryman and Rodewald, 1995) also state that the *ex vivo* studies measured cell surface FcRn in a fixed steady-state distribution and do not take into consideration the rapid rate of receptor recycling which could increase uptake efficiency.

We aimed to examine the contribution of cell surface FcRn in IgG uptake with non-specific pinocytosis and endosomal FcRn binding in our *in vivo* model using the high and low FcRn-binders (N434A and H435A, respectively). The variants were administered at the duodenum in pH conditions of 6.0, 7.4, and 8.0.

The majority of uptake into the cell was by pinocytosis and is pH-independent as shown by comparable serum levels of the FcRn-binding variant, N434A, at pH 7.4 and 8.0. However, when pH conditions were lowered to 6.0, twice the amount of N434A was seen in the serum. This was due to extracellular conditions being optimal for cell surface binding and increased uptake, occurring along with pinocytosis, thereby increasing the number of FcRn–IgG complexes within the cell. Unbound non-FcRn binding IgGs (H435A) were also taken up by the cell but are degraded by lysosomes as they are not protected by FcRn. When the FcRn high-binder, IgG1 N434A, was administered at the ileum (at pH 6.0), trace levels were observed in the serum confirming the need for the presence of FcRn for IgGs to pass into the serum (data not shown). These pH-dependent changes of IgG trancytosis were not seen in the previous *ex vivo* study (Benlounes et al., 1995); however, *ex vivo* tissue preparation and manipulation may have affected the cell surface receptor density. Immuno-histochemical staining in the present study agreed with the *in vivo* data in that the greatest staining on the cell surface of enterocytes was observed at pH 6.0, with less at pH 7.4, and no staining with the FcRn low-binder, H435A. The immuno-histochemical stainings were semi-quantitative and are only used as additional evidence to support the observation that FcRn is present. The possibility of pinocytosis being increased due to the lowering of the local pH is also addressed by the pH study being repeated with administration at the ileum where transcytosis is much lower due to much less FcRn present. If an increase in pinocytosis was caused by a decrease in pH, then there should be also be a difference at this location, albeit much less. However, pH did not have any effect and transcytosis was remained low in all conditions.

This pH-dependent increase lends further evidence that FcRn is also present on the cell surface of the enterocytes in neonatal mammals.

Unlike oral gavage or feed-tubes which do not allow for bypass of the harsh acidic conditions of the stomach, the technique of direct administration into either the duodenum or ileum allows for confidence that the correct dose of IgG is at the appropriately tested site for uptake having not been degraded by stomach acids and proteases. One limitation of the suckling rat pup model as an *in vivo* model is during the early postnatal life, the number of endocytic complex system (including coated pits, apical invaginations, coated vesicles, vesicles, tubules, and endosomes as well as FcRn) is highly increased to facilitate the absorption of the macromolecules and IgG ingested from maternal milk, and this is significantly reduced after weaning (Baba et al., 2002); this does explainwhy low levels of the non-FcRn binding variant, H435A, were still seen in the serum. However, it is advantageous over the Ussing chamber as viable animals consist of fully functional circulatory and lymphatic systems which are essential to IgG uptake from the small intestine.

In summary, intracellular and cell surface FcRn contributes to IgG uptake into the serum. Increased serum levels of IgG variants correlated with their FcRn off-rates at pH 7.4. Serum levels were greatest when administered at the duodenum; the region with the greatest amount of FcRn. Uptake into the cells for transcytosis could be further potentiated when local environments were buffered at pH 6.0 to allow for greater binding of IgGs to FcRn molecules on the cell surface. These results encourage future studies to evaluate therapeutic IgGs which contain an Fc portion to promote the advancement of orally available protein therapies.

## AUTHOR CONTRIBUTIONS

*Participated in research design:* Philip R. Cooper, Connie M. Kliwinski, Gordon D. Powers, John R. Mabus, Haimanti Dorai, Jill Giles-Komar, Pamela J. Hornby.

*Conducted experiments:* Philip R. Cooper, Connie M. Kliwinski, Robert A. Perkinson, Edwin Ragwan, John R. Mabus.

*Contributed new reagents or analytic tools:* Pamela J. Hornby.

*Performed data analysis:* Philip R. Cooper, Robert A. Perkinson, Edwin Ragwan.

*Wrote or contributed to the writing of the manuscript:* Philip R. Cooper, Connie M. Kliwinski, Robert A. Perkinson, Pamela J. Hornby.

## Conflict of Interest Statement

The authors declare that the research was conducted in the absence of any commercial or financial relationships that could be construed as a potential conflict of interest.

## ABBREVIATIONS

FcRn: neonatal Fc receptor
IgG: immunoglobulin G
WT: wild type
